# Associations between T-cell traits and narcolepsy type 1: new insights from a Mendelian randomization study

**DOI:** 10.3389/fneur.2024.1444753

**Published:** 2024-10-31

**Authors:** Shiqin Chen, Tian Lv, Zongshan Li, Gonghua Pan, Yiqiao Chen, Xingwang Zhao, Lisan Zhang

**Affiliations:** ^1^Department of Neurology, Yuhuan Second People’s Hospital, Yuhuan, China; ^2^Department of Neurology, Zhuji Affiliated Hospital of Wenzhou Medical University, Zhuji, China; ^3^Department of Neurology, Xuanwu Hospital of Capital Medical University, Beijing, China; ^4^Department of Neurology, Qingtian People’s Hospital, Lishui, China; ^5^Key Laboratory of Precision Nutrition and Food Quality, Department of Nutrition and Health, China Agricultural University, Beijing, China; ^6^Center for Sleep Medicine, Sir Run Run Shaw Hospital, Zhejiang University School of Medicine, Hangzhou, China

**Keywords:** narcolepsy type 1, herpesvirus entry mediator, CD4^−^ CD8^−^ T-cells, Mendelian randomization, T-cells

## Abstract

**Background:**

Narcolepsy type 1 (NT1) is primarily caused by a malfunctioning immune system in which T-cells damage the hypothalamus. To elucidate the causal relationships between biomarkers in T-cells and NT1, we employed Mendelian randomization (MR) analysis.

**Methods:**

We conducted a two-sample MR analysis utilizing genetically predicted T-cell traits to examine their effects on NT1. Genome-wide association study summary data were extracted from studies by Valeria (3,757 participants) for 211 T-cell traits, Ollila (6,073 cases and 84,856 controls) for NT1. The MR analysis was executed at two threshold levels. Inverse variance weighted, Wald ratio, weighted median, and MR-Egger regression methods were used for the MR analysis. Odds ratios (ORs) were calculated, and heterogeneity tests, as well as pleiotropy tests, were conducted.

**Results:**

After Bonferroni correction at the significant level (*p* < 1.18 × 10^−4^), a higher ratio of naive CD4^−^ CD8^−^ T-cells was identified as a risk factor for NT1 (OR = 10.50; 95% CI: 6.98, 15.90, *p* = 3.89 ×10^−29^). Conversely, CD4 on HLA DR^+^ CD4^+^ T cells (mean fluorescence intensity, MFI) exhibited a negative correlation with NT1. At nominally significant levels (*p* < 0.05) for both threshold levels, HVEM (herpesvirus entry mediator) on naive CD8^+^ T cells (MFI) was suggested as a protective factor for NT1. Additionally, a higher ratio of CD25^++^ CD45RA^−^ CD4 not regulatory T cells, CD127 on CD45RA^−^ CD4 not regulatory T cells (MFI), CD127 on CD28^+^ CD4^+^ T cells (MFI), CD3 on HLA DR^+^ T cells (MFI), and CD3 on HLA DR^+^ CD4^+^ T cells (MFI) were suggested as risk factors for NT1.

**Conclusion:**

This study confirmed the causal effects of CD4^+^ and CD8^+^ T-cells on NT1 and found several novel T-cell-related characteristics.

## Introduction

Narcolepsy type 1 (NT1) is a neurological disorder primarily attributed to immune system dysfunction (1, 2). More specifically, the malfunction is believed to originate from T-cells (3–5), where an aberrant immune response leads to progressive damage to the hypothalamus (6). The hypothalamus plays a crucial role in regulating the sleep-wake cycle and sleep (7). However, in individuals with NT1, malfunctioning T-cells attack and destroy healthy cells in the hypothalamus, disrupting its normal functioning (5); this leads to a significant decrease in orexin. Patients with NT1 face challenges in the workplace, school, and home settings; they may be labeled as “lazy” and are at greater risk for psychological disorders, obesity, social problems, and traffic accidents (8–11).

Extensive research has been conducted on the roles of CD4 and CD8 T-cells in narcolepsy (4, 11, 12). Moreover, three studies have characterized disease-associated changes in the immune system of individuals with NT1 and have assessed whether NT1 patients exhibit a distinct immune signature involving specific T-cell subsets (13–15). Specifically, Hartmann et al. (13) found that NT1 patients have higher proportions of naive CD4 and CD8 T cells, accompanied by fewer respective effector memory T cells; similarly, Viste et al. (4) observed that NT1 is associated with lower levels of effector memory CD4^+^ T cells, but found inconsistency regarding an increase in naive CD4 T cells; Pedersen et al. (12) noted a higher frequency of autoreactive CD8^+^ T cells in NT1 patients compared to healthy controls, suggesting their potential significance in NT1 development; However, while Lecendreux et al. (15) and Moresco et al. (14) reported no significant differences in the total frequency of CD4 and CD8 T cells between NT1 patients and controls, they observed increased expression of CD25 and CD69 on CD4 T cells. Nevertheless, the limited number of narcolepsy cases makes it difficult to control confounding factors during clinical studies, leading to inconsistent results. Moreover, the role of other immune cell types in NT1 pathogenesis remains uncertain. Mendelian randomization (MR) could be useful to overcome these limitations. The MR approach uses genetic variants to evaluate the causal associations between exposure and outcome variables (16). MR studies, being less susceptible to confounding and reverse causality bias (17), suggest that when applied to large datasets, MR analysis could serve as a promising approach to explore novel T-cell biomarkers influencing the risk for NT1.

We hypothesized that the expression profile of T-cell surface molecules (such as CD4^+^ or CD8^+^ T cells) and molecular functional changes due to single-nucleotide polymorphisms (SNPs) act as regulatory factors influencing T-cell-mediated immune abnormalities and damage to hypothalamic orexin neurons. We used a two-sample MR approach to test this hypothesis. T-cell data from a cohort of 3,757 individuals of European descent were analyzed, along with data for 6,073 NT1 patients and 84,856 healthy controls from a genome-wide association study (GWAS).

## Methods

### Study design

**Figure 1:**
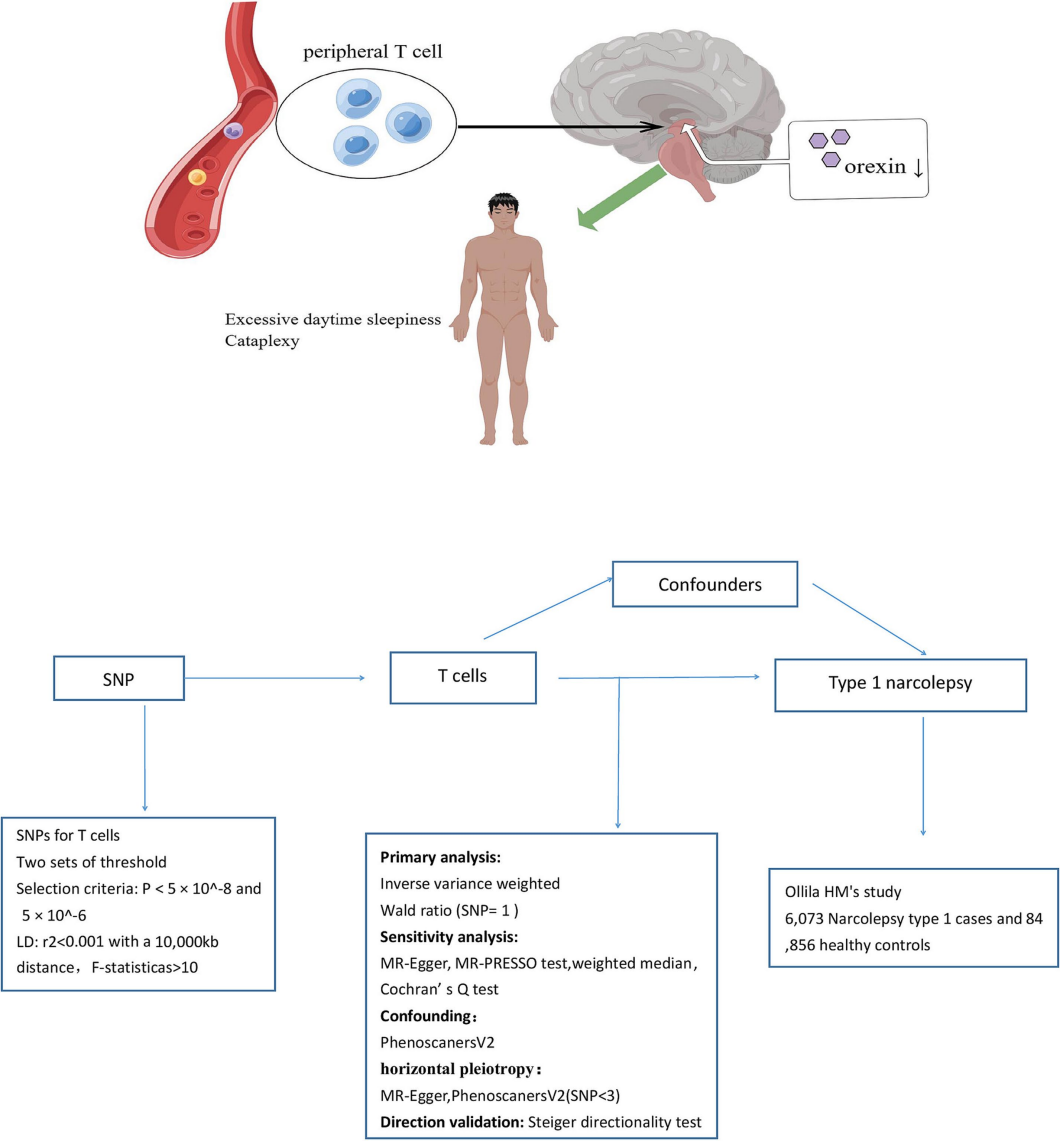
Study design schematic.

We examined the causal relationships between T-cell traits and NT1 risk using a two-sample MR approach. Figure 1 provides an overview of the study design. The validity of the instrumental variable (IV) in our MR analysis depended on three assumptions: there is a significant association between genetic variation (the IV) and T-cell traits (exposure variable), genetic variation is independent of known and unknown confounding factors, and the associations between genetic variation and NT1 are mediated only by T-cell traits. To obtain the necessary data, we primarily relied on independent GWAS datasets. Within the MR framework, SNPs were utilized to evaluate the causal associations between T-cell traits and NT1.

### Data sources

Exposure data were obtained from the GWAS “Complex genetic signature of immune cells underlies autoimmunity and informs therapy,” by Orrù et al. (18). Their dataset encompasses approximately 22 million variants associated with 731 immune cell traits. These traits were observed in a cohort of 3,757 individuals of European descent who underwent profiling via flow cytometry. The 731 immune cell traits included 118 absolute cell counts, 389 mean fluorescence intensities related to surface antigens, 32 morphological parameters, and 192 relative cell counts (Supplementary Table S1). We extracted a total of 211 T-cell traits (Supplementary Table S2) as exposure data. These traits included various T-cell subtypes [double-positive, double-negative (DN), CD4, and CD8 T-cells], T-cell maturation stages (central memory, effector memory, and terminally differentiated), a regulatory T-cell panel, and the expression levels of cell markers in different T-cells.

The genetic data for NT1 were sourced from the latest and largest GWAS conducted by Ollila et al. (5), which involved 6,073 cases and 84,856 controls. This GWAS predominantly included individuals of European descent (~88%) across 13 cohorts worldwide. The study successfully identified 13 independent risk loci, seven of which had not been previously reported. Detailed information about the cohorts is available in Supplementary Table S3. Since approximately 88% of the participants were of European ancestry, we considered the summary data from this GWAS to be representative of the European population for MR analysis.

In summary, using the most comprehensive and up-to-date GWAS datasets focused on T-cell traits, we investigated the causal relationships between T-cell traits and NT1.

### SNP selection

To validate the causal relationships between T-cell traits and NT1, we implemented rigorous quality-control procedures to select suitable IVs. Initially, we identified SNPs significantly associated with T-cell traits that surpassed the threshold for genome-wide significance (*p* < 5 × 10^−8^). As some traits had fewer than three independent SNPs, we concurrently applied a filtering threshold of 5 × 10^−6^. Two sets of threshold criteria were employed for separate analysis. Then we grouped the SNPs based on the European 1,000 Genomes Project reference panel, using an *r*^2^ value <0.001 and a distance >10,000 kb to identify independent SNPs. We also determined the minor allele frequency of each palindromic SNP; if the frequency was <0.30, we classified the SNP as palindromic.

To ensure that the effect of each IV on the exposure and outcome variables corresponded with the positive-strand alleles, we harmonized the T-cell trait and NT1 data. Then we utilized the *F*-statistic (beta²/se²) to estimate the strength of the associations with exposure traits. If the *F*-statistic was <10 in MR analyses, there was no significant association between the IV and T-cell traits, suggesting potential bias.

### Statistical analysis

#### MR analysis

The causal effect of exposure variables on the outcome variables was evaluated using various MR methods. The inverse variance weighted (IVW) method was used when there was more than one SNP, whereas the Wald ratio (WR) was employed when only one SNP was available. The IVW method served as the primary approach and was employed when all SNPs were available and valid (19). This method ensures consistent estimation of causal relationships between exposure and outcome variables, provided that the genetic variation satisfies the model assumptions and is unaffected by pleiotropy (20). Odds ratios (ORs) were calculated, and the outcome variables were dichotomous.

#### Sensitivity analysis

When more than three SNPs were available, sensitivity analyses were carried out using a number of MR approaches, including weighted median, MR-Egger regression, and Mendelian randomization-pleiotropy residual sum and outlier (MR-PRESSO), which all make different assumptions affecting the statistical power. The weighted median approach gives consistent estimates provided that more than half of the weights are derived from valid SNPs (21). When all genetic variants exhibit pleiotropic effects, MR-Egger analysis is capable of adjusting for pleiotropy and making causal inferences (22). Heterogeneity was assessed using Cochran’s *Q* test (23). We employed the MR-PRESSO approach to identify and remove SNPs with horizontal pleiotropic outliers to minimize pleiotropic effects on causal estimates (21). Outliers significant at *p* < 0.05 were excluded, and the remaining SNPs were subjected to IVW analysis. In addition, the presence of pleiotropy in individual SNPs was determined based on the MR-Egger regression intercept, with *p*-values >0.05 indicating that there was no horizontal pleiotropy (24). Furthermore, leave-one-out analysis was conducted to investigate the impact of each SNP on pleiotropy. For exposure variables with fewer than three SNPs, we conducted pleiotropy analysis by searching the PhenoScanner database for additional traits associated with the identified SNPs in previous GWASs (25). Finally, the MR Steiger directionality test was performed to confirm that the exposure caused the outcome in cases where the result was statistically significant (26). We graphed the relationships between T-cell traits and NT1 to facilitate interpretation of our findings.

The Two-Sample MR (27) and Mendelian randomization (28) packages were utilized to perform all analyses in the R environment (ver. 4.2.2; R Development Core Team, Vienna, Austria). The results are reported as ORs and 95% confidence intervals (CIs). Given the repeated comparisons of T-cell traits with each outcome, *p*-values underwent Bonferroni correction, with the significance threshold set at 0.05/422 (1.18 × 10^–4^). Additionally, results with *p*-values <0.05 were considered nominally significant.

## Results

### T-cell traits

Supplementary Table S4 (SNP selection at a threshold of 5 × 10^−8^) and Supplementary Table S5 (threshold of 5 × 10^−6^) provide more information on the 211 T-cell traits analyzed in this study, including beta coefficients, standard errors, and *p*-values for the SNPs. The *F*-values ranged from 20.90 to 3161.45, indicating a lack of bias.

### Causal relationships between T-cell traits and NT1

We conducted a comprehensive MR study utilizing genetically predicted T-cell traits to examine their effects on NT1. The MR analysis was conducted at different threshold levels. Detailed MR results regarding the associations between T-cell traits and NT1 in each dataset can be found in Supplementary Tables S6, S7.

**Figure 2:**
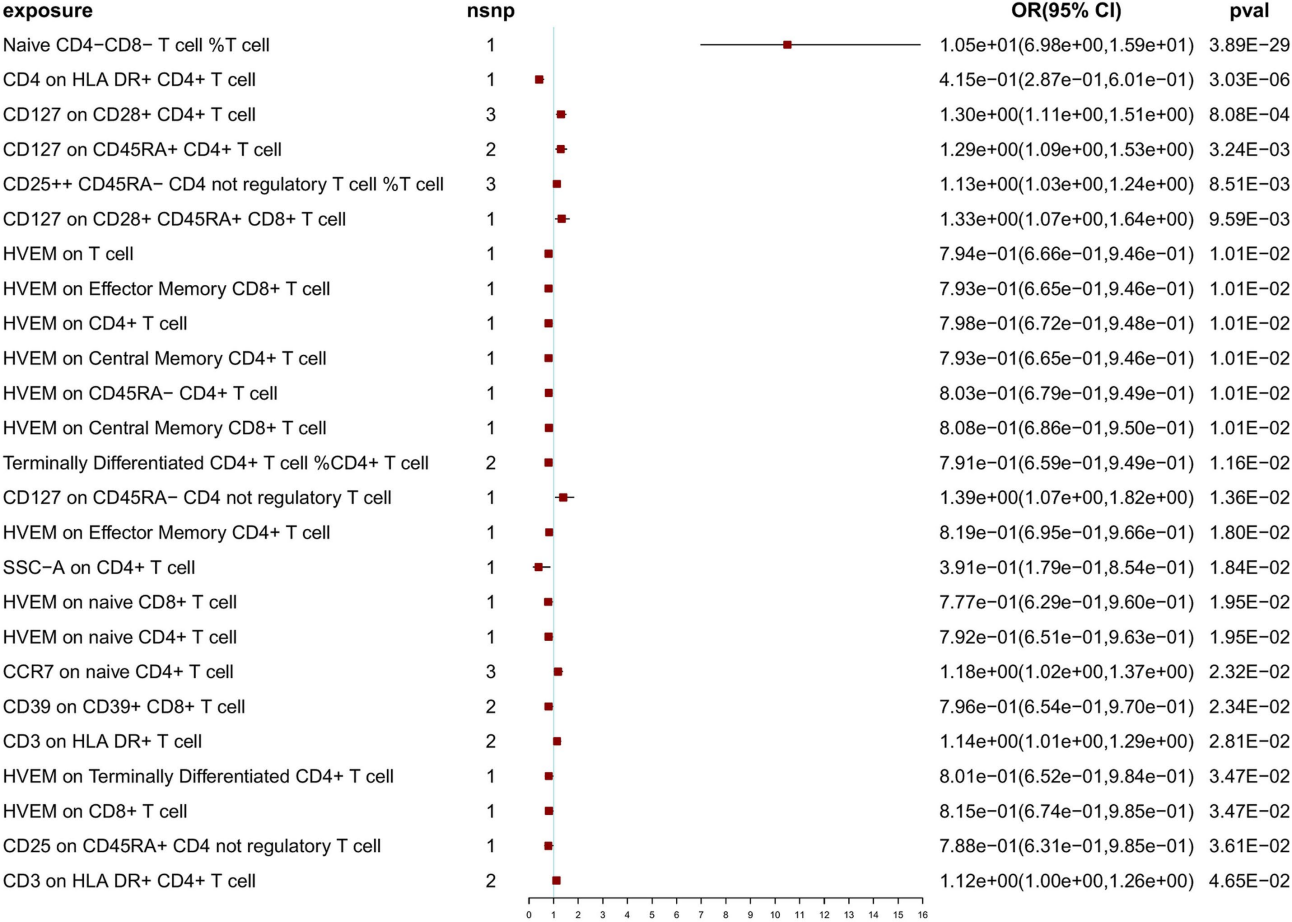
Primary Mendelian randomization analysis results at 5 × 10^−8^ SNP selection threshold.

At a threshold of 5 × 10^−8^ for SNP selection in the MR analysis, after Bonferroni correction (*p* < 1.18 × 10^–4^), we identified 2 significant associations with NT1 (naive CD4^−^ CD8^−^ T cell %T cell: OR = 10.50; 95% CI: 6.98, 15.90, *p* = 3.89 × 10^−29^; CD4 on HLA DR^+^ CD4^+^ T cell: OR = 4.15 × 10^−1^; 95% CI: 2.87 × 10^−1^, 6.01 × 10^−1^, *p* = 3.03 × 10^−6^), and 23 nominally significant associations (Figure 2). Notably, among the exposure variables, 3 T-cell traits each had three SNPs, while the remaining variables had fewer than 3 SNPs each. When SNPs ≥ 3, to ensure the robustness of our findings, sensitivity analyses using the weighted median test and MR-Egger regression were also performed (Supplementary Table S8). IVW, weighted median, and MR-Egger tests consistently produced results in the same direction. Based on this consistency, we considered the findings to be robust and reliable, and generated a scatterplot (Supplementary Figures S1–S3). The MR-PRESSO test was not feasible due to the limited number of SNPs. According to the Cochran’s *Q* test, there was no significant heterogeneity (*p* > 0.05). The *p*-value (>0.05) obtained from the MR-Egger intercept analysis indicated the absence of horizontal pleiotropy. To further evaluate our findings, we examined forest and funnel plots (Supplementary Figures S1–S3). In addition, leave-one-out analysis demonstrated the robustness of our primary outcome data. Finally, the Steiger directionality test was performed, confirming that all significant results were in line with the expected exposure-to-outcome direction (Supplementary Table S8). For exposure variables with fewer than three SNPs. To investigate potential pleiotropic effects and additional associated traits identified in previous GWASs, the PhenoScanner database was utilized. Findings of the pleiotropy analysis are provided in Supplementary Table S9; no significant effects of pleiotropy were found.

**Figure 3:**
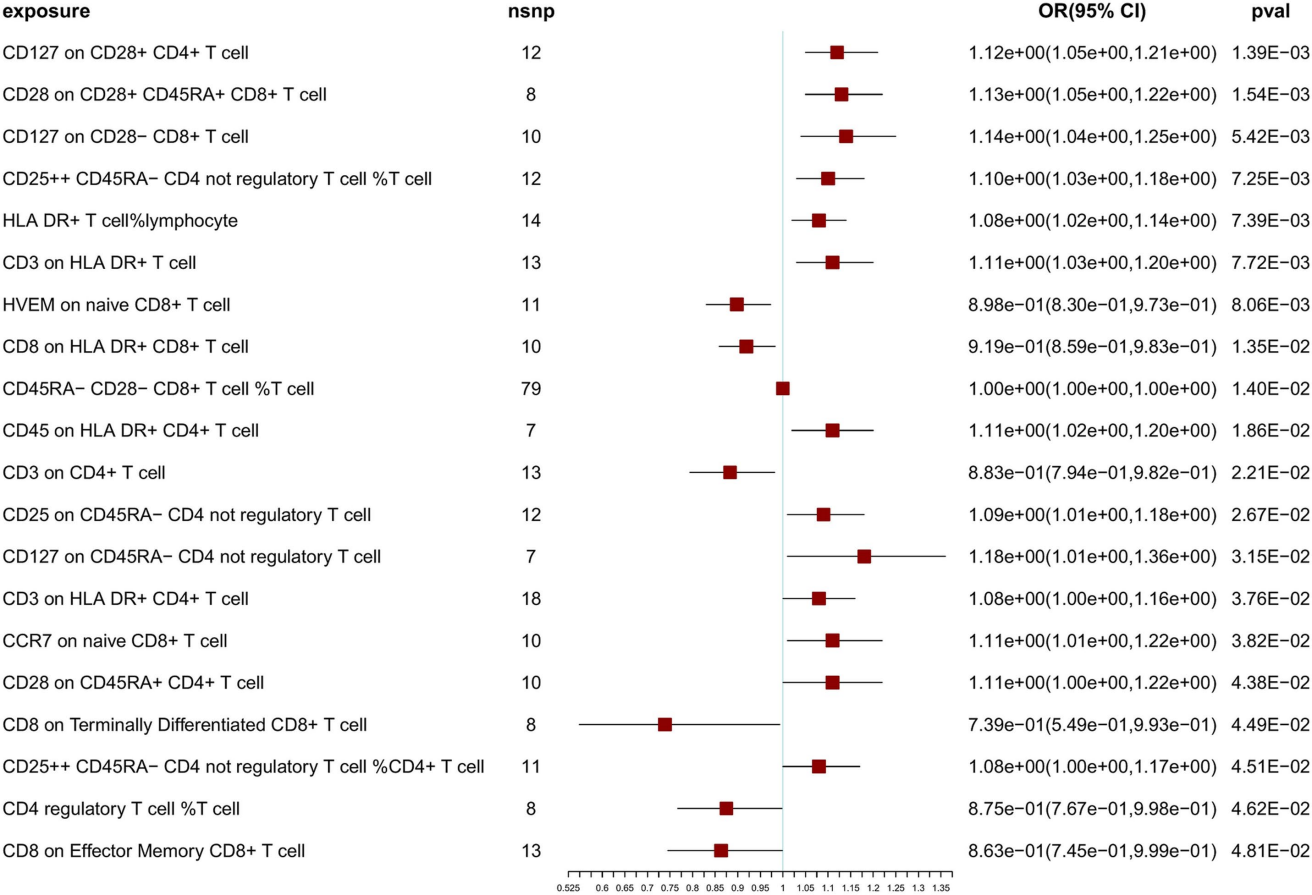
Primary Mendelian randomization analysis results at 5 × 10^−6^ SNP selection threshold.

To further confirm the correlation between T-cell traits and NT1, we relaxed the SNP selection threshold. At a threshold of 5 × 10^−6^ for SNP selection in the MR analysis, after Bonferroni correction, we observed no significant associations with NT1, but identified 20 nominally significant associations (Figure 3). Sensitivity analyses were also conducted using multiple methods (Supplementary Table S10). IVW, weighted median, and MR-Egger tests were employed to assess the reliability of results of the MR analysis, and a scatterplot was generated (Supplementary Figures S4–S23). Cochran’s *Q*-derived *p*-values were all >0.05, except for the estimates of CD8 on Effector Memory CD8^+^ T cell and CD8 on Terminally Differentiated CD8^+^ T cell effects on NT1. An outlier, rs17201560 (CD8 on Effector Memory CD8^+^ T cell impact on NT1), and two outliers, rs114373132 and rs960502 (CD8 on Terminally Differentiated CD8^+^ T cell impact on NT1), were detected through MR-PRESSO. Following its exclusion, the analysis was reiterated, and the results were robust, with no significant heterogeneity (Supplementary Table S10). All *p*-values (>0.05) derived from the MR-Egger intercept analysis indicated the absence of horizontal pleiotropy. We also examined the forest and funnel plots (Supplementary Figures S4–S23). Leave-one-out analysis revealed that the estimates remained unbiased by a single SNP, indicating no violation of estimates and highlighting the robustness of our primary findings. The Steiger directionality test indicates the correct exposure to outcome direction (Supplementary Table S10).

It is important to note that, at both threshold levels of 5 × 10^−8^ and 5 × 10^−6^, we simultaneously identified 6 nominally significant associations with NT1 (Table 1). We consider these results to be robust. However, it is essential to recognize that this study falls under exploratory research, and we do not exclude the possibility of other potential outcomes at different threshold levels.

**Table 1:** Overlapping results of T-cell traits nominally significant associations with NT1 at 5 × 10^−8^ and 5 × 10^−6^ SNP selection thresholds.

T-cell traits’ effects on NT1: primary MR analyses at a threshold of 5 × 10^−6^ for SNP					
Exposure	Outcome	*n*SNP	Method	OR (95% confidence intervals)	*p*val
CD25^++^ CD45RA^−^ CD4 not regulatory T cell %T cell	NT1	12	Inverse variance weighted	1.10 × 10^0^ (1.03 × 10^0^, 1.18 × 10^0^)	7.25 × 10^3^
CD3 on HLA DR^+^ T cell	NT1	13	Inverse variance weighted	1.11 × 10^0^ (1.03 × 10^0^, 1.20 × 10^0^)	7.72 × 10^−3^
CD3 on HLA DR^+^ CD4^+^ T cell	NT1	18	Inverse variance weighted	1.08 × 10^0^ (1.00 × 10^0^, 1.16 × 10^0^)	3.76 × 10^−2^
HVEM on naive CD8^+^ T cell	NT1	11	Inverse variance weighted	8.98 × 10^−1^ (8.30 × 10^−1^, 9.73 × 10^−1^)	8.06 × 10^−3^
CD127 on CD45RA^−^ CD4 not regulatory T cell	NT1	7	Inverse variance weighted	1.18 × 10^0^ (1.01 × 10^0^, 1.36 × 10^0^)	3.15 × 10^−2^
CD127 on CD28^+^ CD4^+^ T cell	NT1	12	Inverse variance weighted	1.12 × 10^0^ (1.05 × 10^0^, 1.21 × 10^0^)	1.39 × 10^−3^

T-cell traits’ effects on NT1: primary MR analyses at a threshold of 5 × 10^−8^ for SNP					
Exposure	Outcome	*n*SNP	Method	OR (95% confidence intervals)	*p*val
CD25^++^ CD45RA^−^ CD4 not regulatory T cell %T cell	NT1	3	Inverse variance weighted	1.13 × 10^0^ (1.03 × 10^0^, 1.24 × 10^0^)	8.51 × 10^−3^
CD3 on HLA DR^+^ T cell	NT1	2	Inverse variance weighted	1.14 × 10^0^ (1.01 × 10^0^, 1.29 × 10^0^)	2.81 × 10^−2^
CD3 on HLA DR^+^ CD4^+^ T cell	NT1	2	Inverse variance weighted	1.12 × 10^0^ (1.00 × 10^0^, 1.26 × 10^0^)	4.65 × 10^−2^
HVEM on naive CD8^+^ T cell	NT1	1	Wald ratio	7.77 × 10^−1^ (6.29 × 10^−1^, 9.60 × 10^−1^)	1.95 × 10^−2^
CD127 on CD45RA^−^ CD4 not regulatory T cell	NT1	1	Wald ratio	1.39 × 10^0^ (1.07 × 10^0^, 1.82 × 10^0^)	1.36 × 10^−2^
CD127 on CD28^+^ CD4^+^ T cell	NT1	3	Inverse variance weighted	1.30 × 10^0^ (1.11 × 10^0^, 1.51 × 10^0^)	8.08 × 10^−4^

NT1, narcolepsy type 1; MR, Mendelian randomization; SNP, single-nucleotide polymorphism; OR, odds ratio.

## Discussion

NT1 may be an autoimmune disorder according to both genetic and immunological evidence. The involvement of the MHC class II DQB1*06:02 allele and a specific polymorphism in the TCR α chain locus confirms the genetic basis of the condition (29, 30). Moreover, several additional lines of evidence underscore the role of a T-cell-mediated immune mechanism in the destruction of orexin neurons in individuals with NT1, with CD4^+^ and CD8^+^ cells being particularly important (15, 31–34). The Strengthening the Reporting of Observational Studies in Epidemiology using Mendelian randomization (STROBE-MR) guidelines were followed in this study (35), which confirmed the impact of CD4^+^ and CD8^+^ T-cells on NT1 and also yielded new insights.

In our study, at the significant level, the ratio of naive CD4^−^ CD8^−^ T cells (naive CD4^−^ CD8^−^ T cell %T cell) showed a positive correlation with NT1, whereas the expression of CD4 on HLA DR^+^ CD4^+^ T cells (mean fluorescence intensity, MFI) exhibited a negative correlation. At nominally significant levels for both threshold levels (5 × 10^−8^ and 5 × 10^−6^), the expression of HVEM (herpesvirus entry mediator) on naive CD8^+^ T cells (MFI) displayed a negative correlation with NT1. Regulatory T cells (Tregs), including the ratio of CD25^++^ CD45RA^−^ CD4 non-regulatory T cells (%T cell), the expression of CD127 on CD45RA^−^ CD4 non-regulatory T cells (MFI), and the expression of CD127 on CD28^+^ CD4^+^ T cells (MFI), showed a positive correlation with NT1. Additionally, T-bet^+^ NK cells (TBNKs), specifically the expression of CD3 on HLA DR^+^ T cells (MFI) and the expression of CD3 on HLA DR^+^ CD4^+^ T cells (MFI), were positively correlated with NT1.

A higher ratio of naive CD4^−^ CD8^−^ T-cells was identified as a risk factor for NT1. This minor subset of T-cells, known as double negative (DN) T-cells, constitutes about 3–5% of all T lymphocytes found in the peripheral blood (36, 37). DN T-cells lack both CD4 and CD8 expression, but maintain CD3 positivity. These DN T cells can be either TCRαβ-positive or TCRγδ-positive, but they do not exhibit natural killer T-cell markers (37). DN T-cells differ from conventional CD4 and CD8 T-cells in that they possess both innate and adaptive immune capabilities (37). DN T-cells with T helper (Th)-like properties are capable of secreting cytokines such as interleukin (IL)-4, IL-17, interferon-γ (IFN-γ), and tumor necrosis factor (TNF)-α, thereby assuming a role analogous to that of CD4 Th cells in cases of infection and autoimmune disease (38–40). Conversely, cytotoxic DN T-cells generate IFN-γ, perforin, and granzyme B, thereby having a cytotoxic effect on hematologic malignancies and solid tumors (41, 42). TCRαβ DN T-cells are also involved in the etiology of autoimmune diseases. The accumulation of DN T-cells is positively correlated with disease activity in conditions such as autoimmune lymphoproliferative syndrome and systemic lupus erythematosus (40, 43). Although increased levels of DN T-cells in narcolepsy have not been demonstrated, patients with narcolepsy display elevated concentrations of cytokines, including IL-4 and IFN-γ (44, 45). When naive CD4^−^ CD8^−^ T-cells in the peripheral blood are activated by antigens and differentiate, they can secrete similar cytokines, such as IL-4 and IFN-γ. This may explain how naive CD4^−^ CD8^−^ T-cells may contribute to the pathogenesis of NT1. Further investigations are warranted to validate this hypothesis.

High expression of HVEM on naive CD8^+^ T cells was suggested as a protective factor for NT1 according to our findings. In recent years, HVEM has emerged as a new immune checkpoint within the tumor necrosis factor receptor superfamily (46). Predominantly found in immune cells, HVEM plays a crucial role in modulating T-cell immune responses by activating both inflammatory and inhibitory signaling pathways (47). HVEM has the ability to bind canonical TNF-related ligands, namely, lymphotoxin-α and LIGHT (lymphotoxin-like), but its unique attribute is its interaction with members of the immunoglobulin superfamily, such as B and T lymphocyte attenuator (BTLA) and CD160 (48). The immunoglobulin superfamily includes two distinct groups of proteins: CTLA-4/CD28/CD80/CD86, which function during the early stages of T-cell activation, and PD-1/PD-L1/PD-L2, which regulate the effector phase of the immune response in peripheral tissues (49). BTLA, a receptor in the immunoglobulin superfamily, acts as an inhibitory receptor in both B- and T-cell signaling pathways (47). Advancements in the field of cancer immunotherapy have been achieved by modulating immune checkpoints, including PD-1 blockade. A case report has suggested a potential link between the onset of narcolepsy and the blockade of immune checkpoints, specifically PD-1, in genetically predisposed individuals (50). As an immune-inhibitory ligand, T cells with high expression of HVEM may serve as a protective factor for NT1. HVEM was classified as an activator drug target in the Pharmaprojects database from Citeline.^1^ However, since it did not meet the strict statistical significance threshold, the exact causal relationship requires further investigation. Nonetheless, we offer meaningful references for future research.

Tregs (the ratio of CD25^++^ CD45RA^−^ CD4 non-regulatory T cells, CD127 on CD45RA^−^ CD4 not regulatory T cells, and CD127 on CD28^+^ CD4^+^ T cells) were suggested as risk factors for NT1. When examining T-cell activation markers, Lecendreux et al. (15) observed higher levels of CD4 T cells positive for CD25 and CD69 in NT1 patients, aligning with our study. This suggests that activated CD4^+^ T-cells, which are not regulatory T-cells, may play a role in the pathogenesis of NT1. However, we did not observe an elevation in CD69, and the association of increased CD127 with NT1 has not been reported.

Other activated T-cells, part of TBNKs, which include CD3 on HLA DR^+^ T-cells and CD3 on HLA DR^+^ CD4^+^ T-cells, were suggested as risk factors for NT1; In contrast, CD4 on HLA DR^+^ CD4^+^ T-cells showed a negative correlation with NT1 in our study. Lippert et al. (51) observed a significant increase in peripheral blood HLA-DR^+^ CD4^+^ T-cells and HLA-DR^+^ CD8^+^ T-cells in NT1 patients compared with healthy controls. This is consistent with CD3 on HLA DR^+^ T-cells and CD3 on HLA DR^+^ CD4^+^ T-cells which we found, providing evidence of T-cell-mediated autoimmunity. However, the role of CD4 on HLA DR^+^ CD4^+^ T cells in NT1 has not been reported in previous studies, warranting further investigation.

### Advantages of study

First, MR offers a substantial improvement over traditional approaches by effectively tackling confounding variables, reverse causality, and the ethical concerns inherent in randomized controlled trials (RCTs) (17). By utilizing genetic instruments like single nucleotide polymorphisms (SNPs), which are strongly linked to exposure factors and sorted randomly based on Mendel’s second law, MR mirrors the design of RCTs (52). This method significantly minimizes confounding and provides a robust framework for evaluating causal relationships between exposure and outcome variables (52). Second, the large-scale datasets from GWAS offer significant statistical power, further enhancing the validity and consistency of our results. Third, we conducted MR analyses at both threshold levels, considering the insufficiency of SNPs at the higher threshold and the potential bias from weak instruments at the lower threshold. This approach yields more cautious results.

Given the latest and largest GWAS (13 cohorts) of NT1, which included 6,073 cases analyzed in this study, a comprehensive data-driven analysis was performed. We consider the MR results to be robust. However, several limitations must be acknowledged. Firstly, the constrained sample size of T-cell traits and the limited number of SNPs pose challenges, as some T-cell traits IV are defined by single SNPs at the threshold of 5 × 10^−8^, thereby limiting statistical power. Secondly, due to the rarity of NT1 cases in publicly available NT1 GWAS, validation was unattainable, necessitating further investigation to confirm our findings. Finally, it is crucial to note that the data primarily originated from individuals of European descent. Thus, additional GWAS studies including participants from other racial backgrounds are required to confirm the generalizability of the results.

## Conclusion

This study provided evidence for a causal relationship between T-cell traits and NT1, and found several novel T-cell-related characteristics: a higher ratio of naive CD4^−^ CD8^−^ T cells, high expression of HVEM on naive CD8^+^ T cells, and other activated T cells. These factors could serve as biomarkers for predicting the development of NT1 and may offer valuable insight into the therapeutic targets. Further research could improve our understanding of NT1 pathogenesis and guide future interventions and prognostic approaches.

## Data Availability

The raw data supporting the conclusions of this article will be made available by the authors, without undue reservation.

## Footnotes

^1^https://pharma.id.informa.com
