# Supplementary material for: Associations between T-cell traits and narcolepsy type 1: new insights from a Mendelian randomization study
Source: Front Neurol. 2024 Oct 31;15:1444753. doi: 10.3389/fneur.2024.1444753 (PMC11560883; doi:10.3389/fneur.2024.1444753)

Supplementary Information

Supplementary Tables

Supplementary Table 1. Characterization of immunophenotypes.

Supplementary Table 2. Information of T-cell traits.

Supplementary Table 3. Descriptions of study cohorts participating in Ollila HM's study.

Supplementary Table 4. SNP selection at a threshold of 5 × 10^-8.

Supplementary Table 5. SNP selection at a threshold of 5 × 10^-6.

Supplementary Table 6. T-cell traits' effects on NT1: Primary Estimate of the MR at a threshold of 5 × 10^-8 for SNP selection.

Supplementary Table 7. T-cell traits' effects on NT1: Primary Estimate of the MR at a threshold of 5 × 10^-6 for SNP selection.

Supplementary Table 8. T-cell traits' effects on NT1: Sensitivity analyses of the MR at a threshold of 5 × 10^-8 for SNP selection.

Supplementary Table 9. Investigating Single-SNP Pleiotropy Analysis with the PhenoScanner V2 Website.

Supplementary Table 10. T-cell traits' effects on NT1: Sensitivity analyses of the MR at a threshold of 5 × 10^-6 for SNP selection.

Supplementary Figures

Part 1. T-cell traits' effects on NT1: MR analyses at a threshold of 5 × 10^-8 for SNP selection.

Figure S1. Scatter, Forest, Funnel, and Leave-one-out plots of genetically predicted CD25++ CD45RA- CD4 not regulatory T cell %T cell effects on NT1.


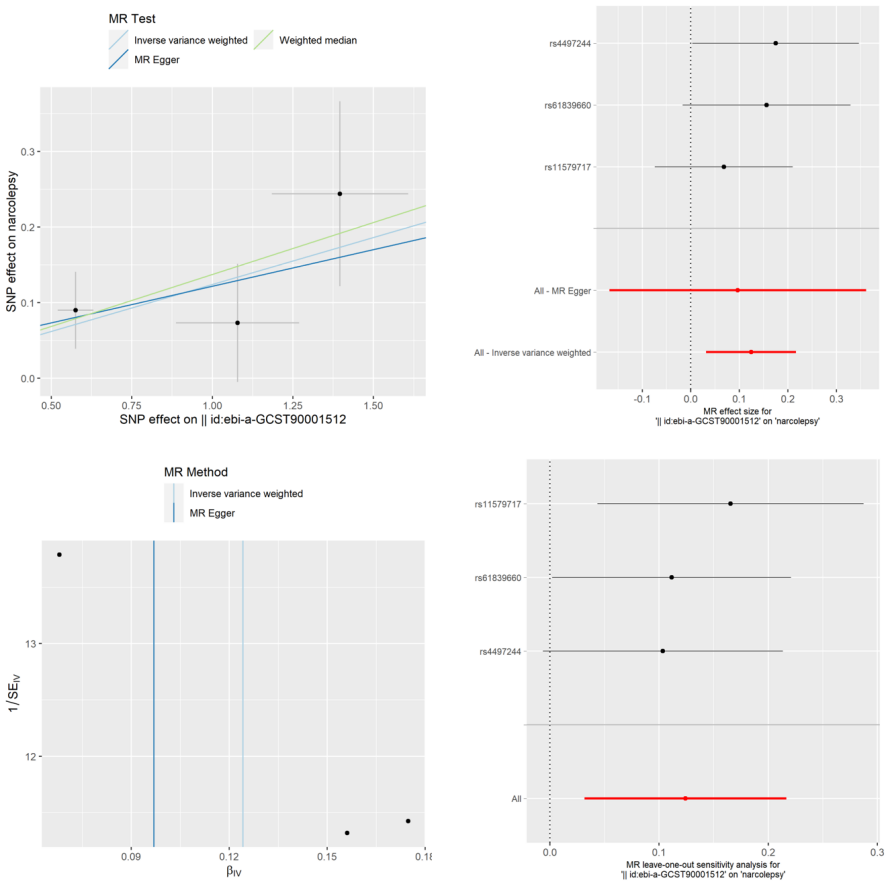


Figure S2. Scatter, Forest, Funnel, and Leave-one-out plots of genetically predicted CCR7 on naive CD4+ T cell effects on NT1.


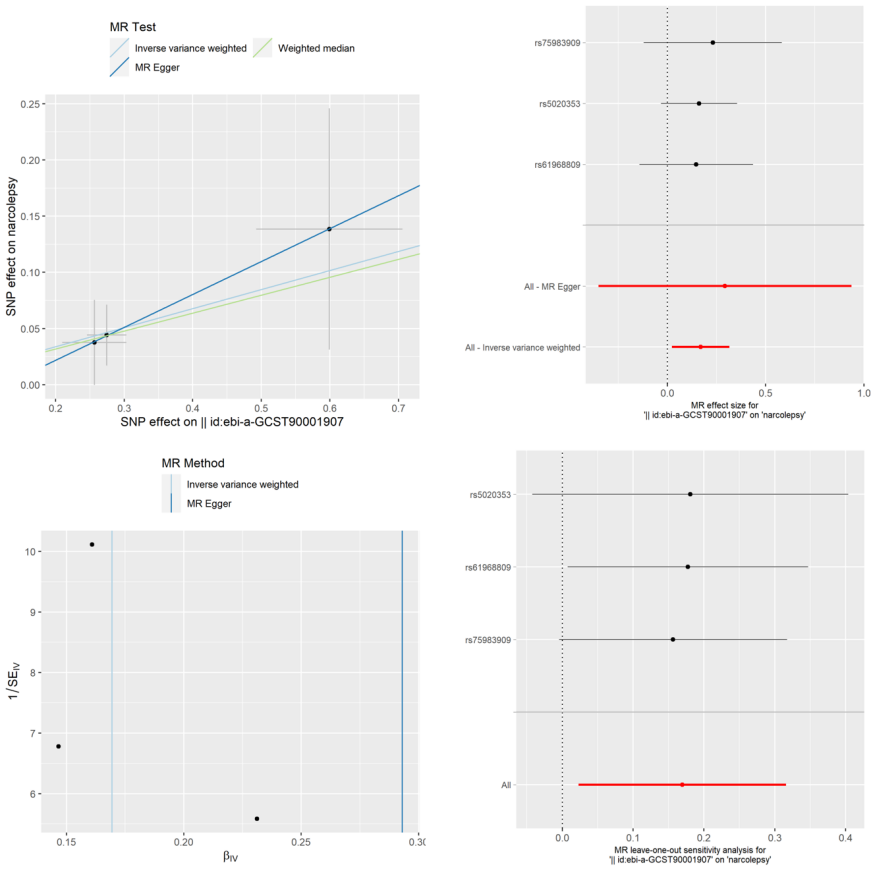


Figure S3. Scatter, Forest, Funnel, and Leave-one-out plots of genetically predicted CD127 on CD28+ CD4+ T cell effects on NT1.


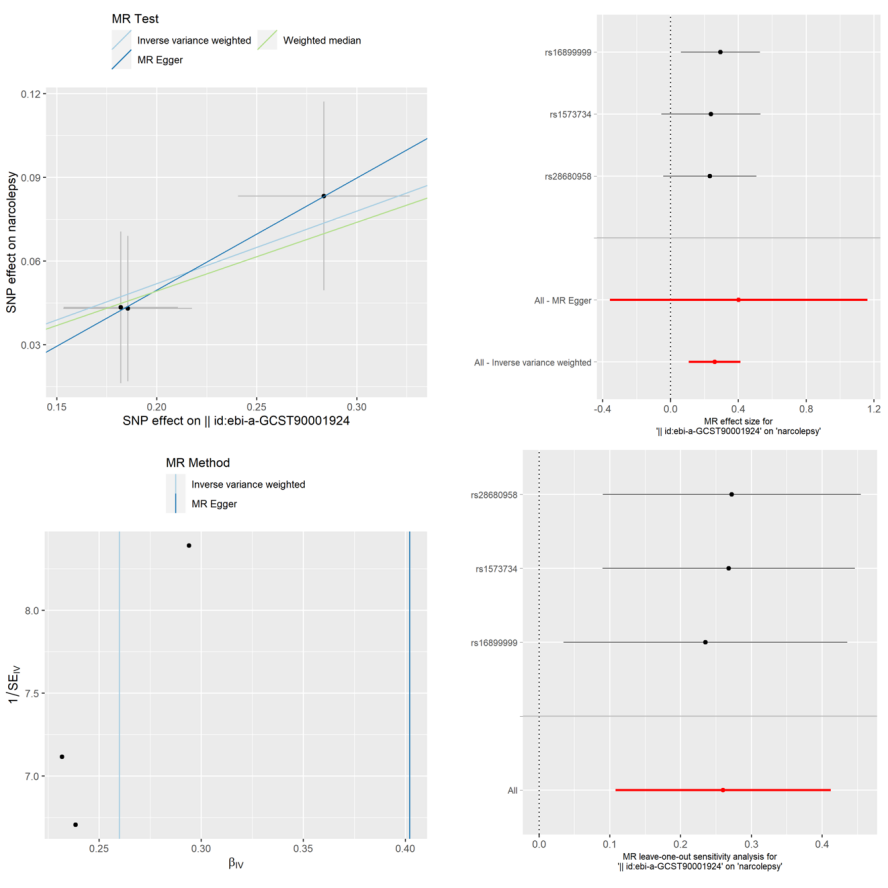


Part 2. T-cell traits' effects on NT1: MR analyses at a threshold of 5 × 10^-6 for SNP selection.

Figure S4. Scatter, Forest, Funnel, and Leave-one-out plots of genetically predicted CD4 regulatory T cell %T cell effects on NT1.


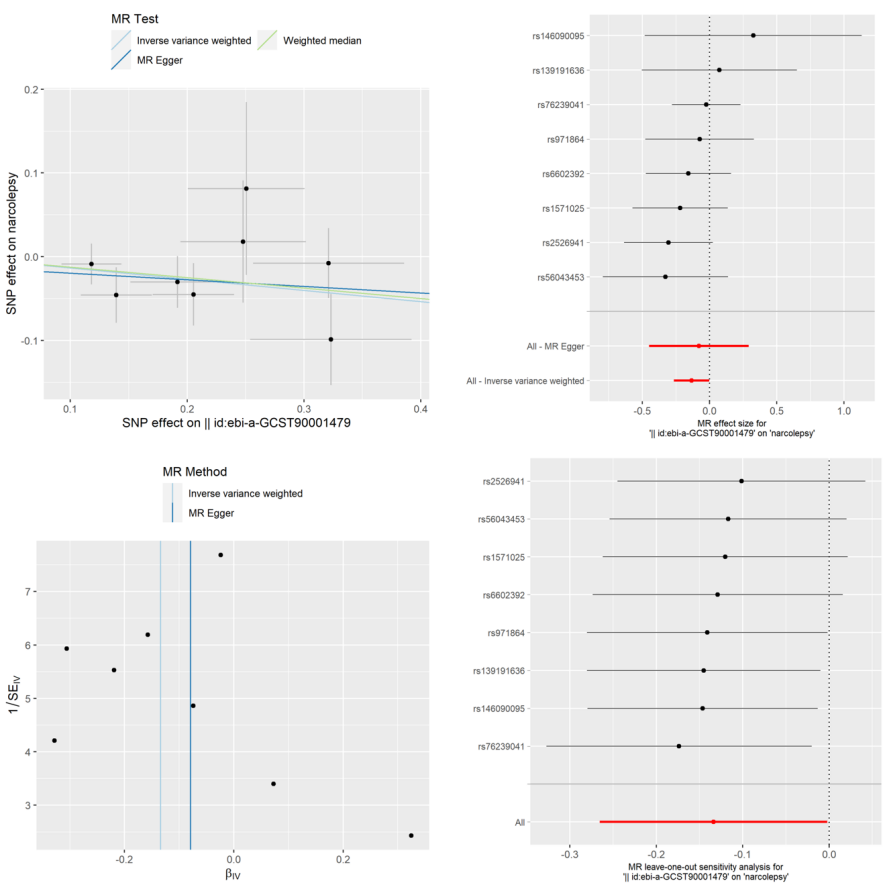


Figure S5. Scatter, Forest, Funnel, and Leave-one-out plots of genetically predicted CD25++ CD45RA- CD4 not regulatory T cell %CD4+ T cell effects on NT1.


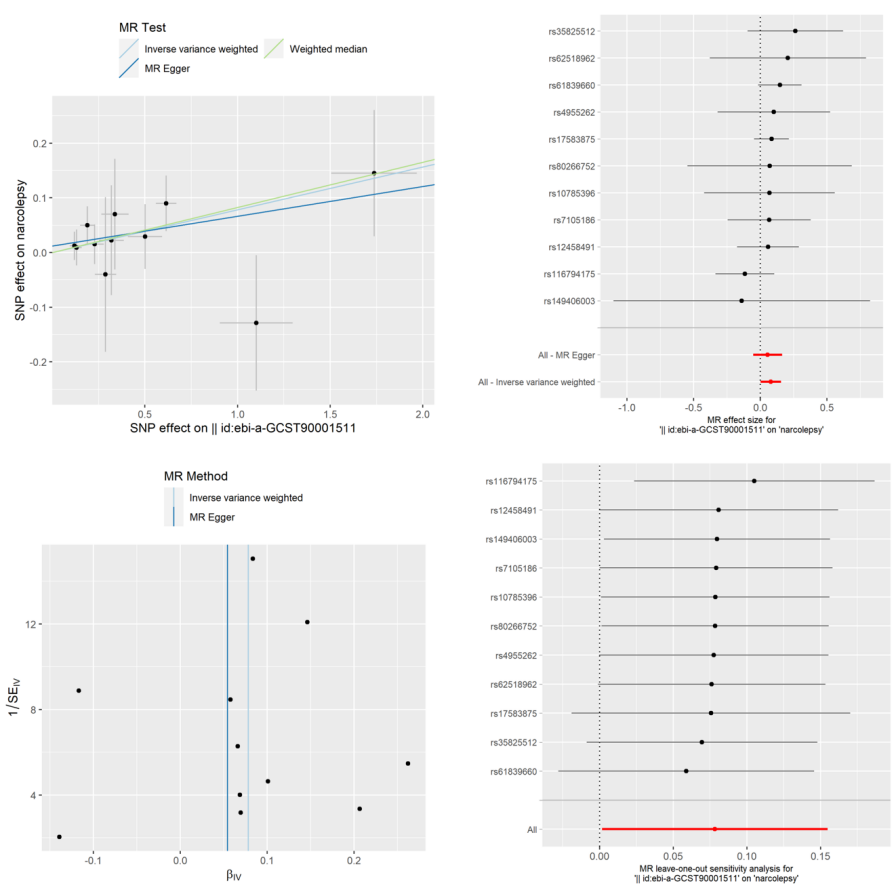


Figure S6. Scatter, Forest, Funnel, and Leave-one-out plots of genetically predicted CD25++ CD45RA- CD4 not regulatory T cell %T cell effects on NT1.


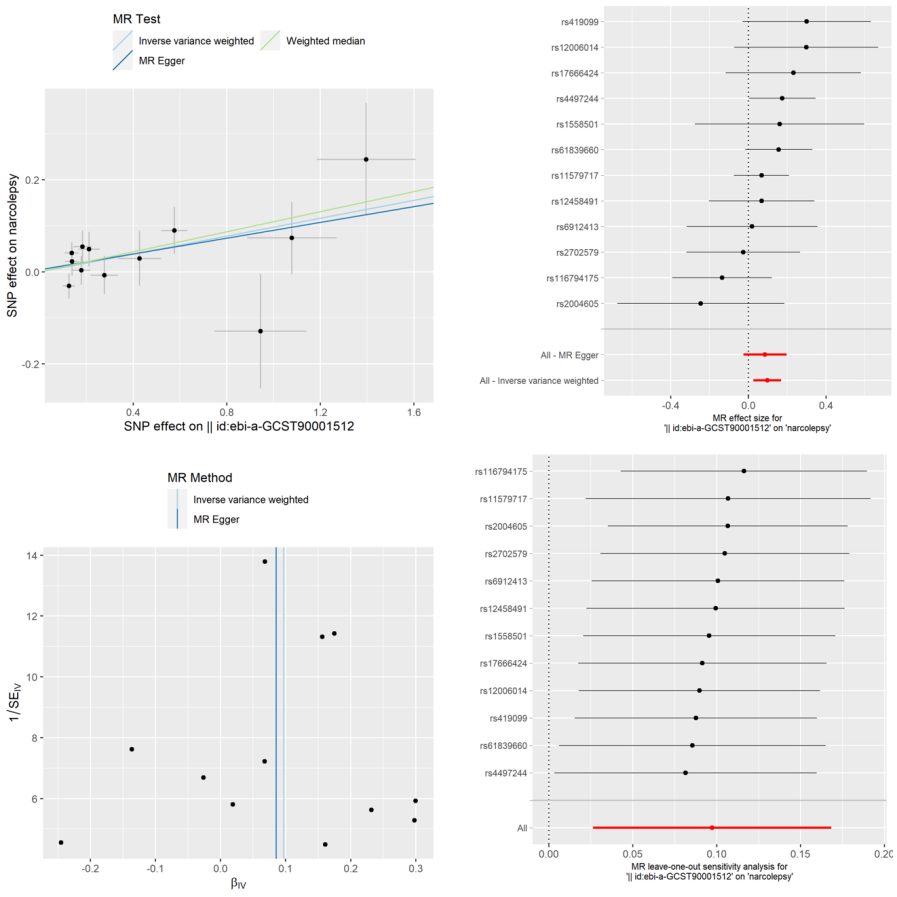


Figure S7. Scatter, Forest, Funnel, and Leave-one-out plots of genetically predicted HLA DR+ T cell%lymphocyte effects on NT1.


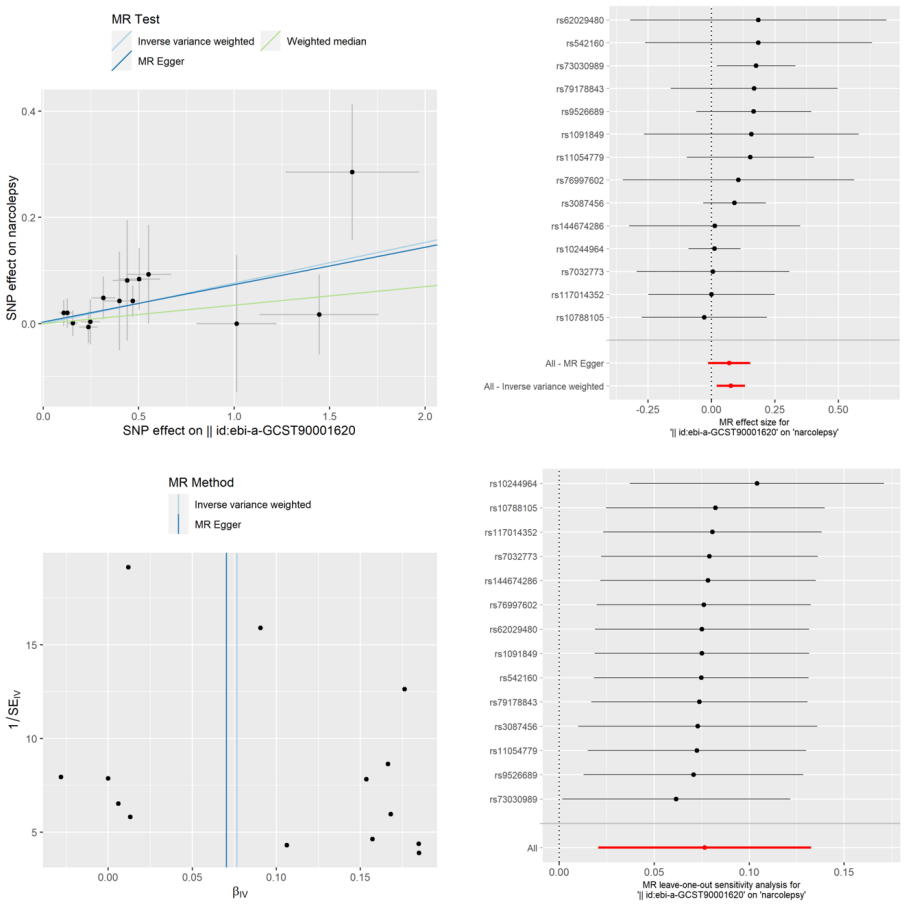


Figure S8. Scatter, Forest, Funnel, and Leave-one-out plots of genetically predicted CD45RA- CD28- CD8+ T cell %T cell effects on NT1.


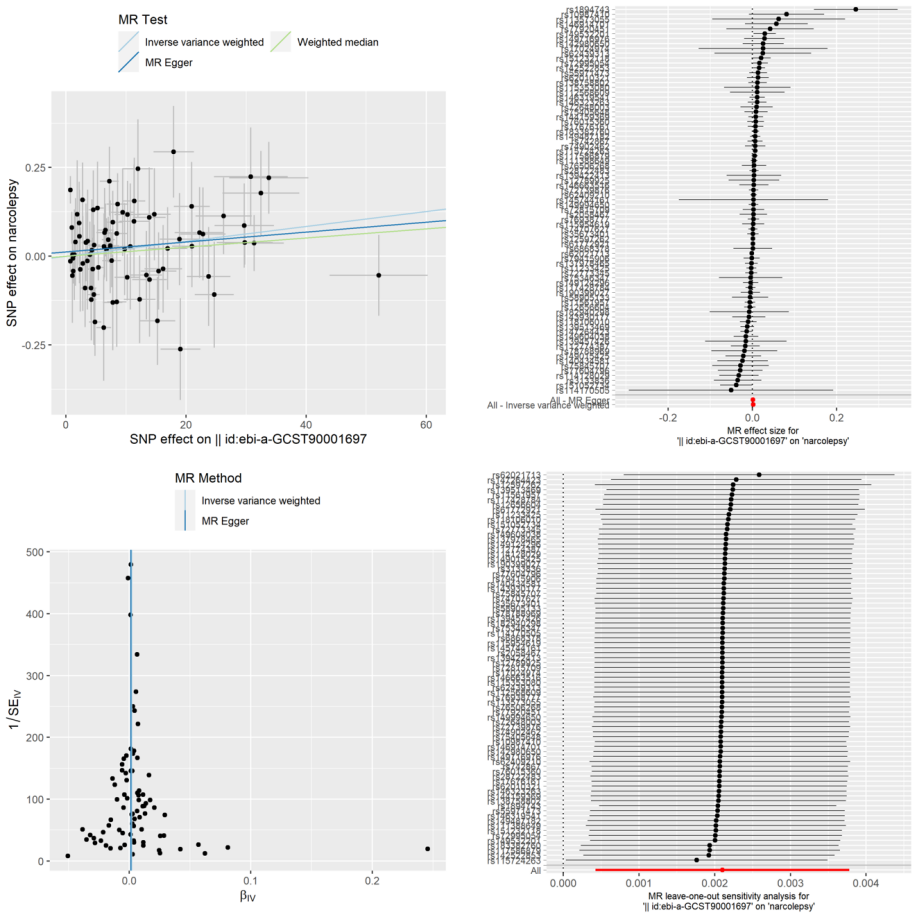


Figure S9. Scatter, Forest, Funnel, and Leave-one-out plots of genetically predicted CD3 on HLA DR+ T cell effects on NT1.


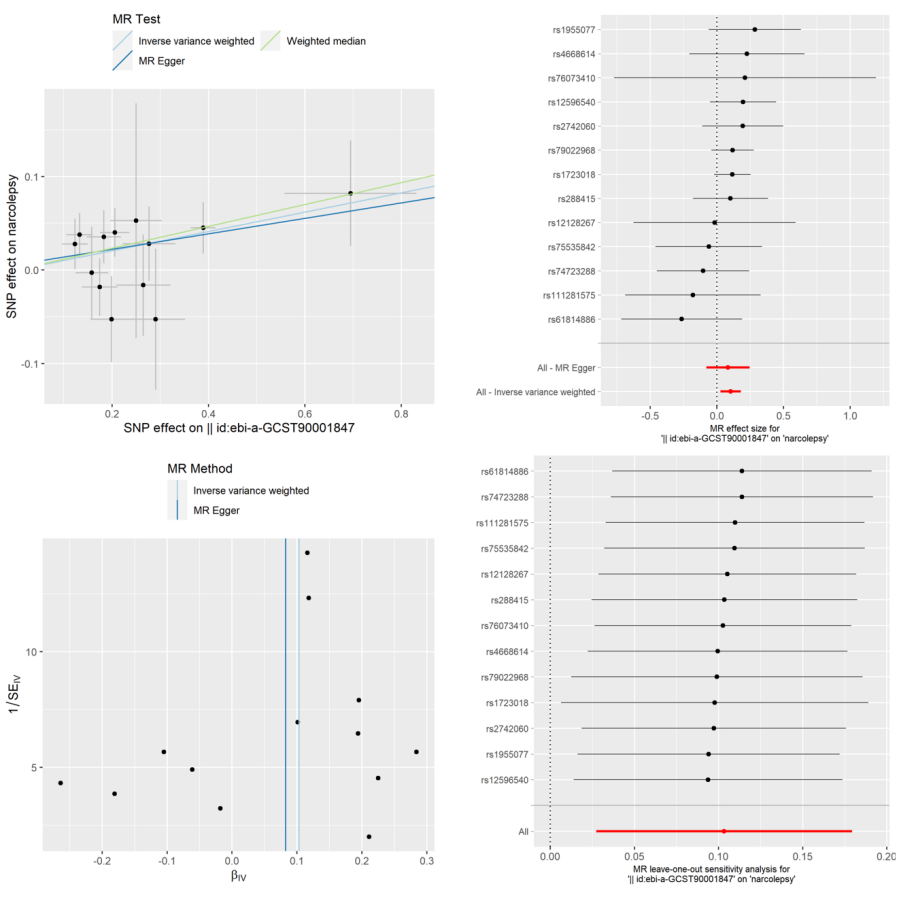


Figure S10. Scatter, Forest, Funnel, and Leave-one-out plots of genetically predicted CD3 on HLA DR+ CD4+ T cell effects on NT1.


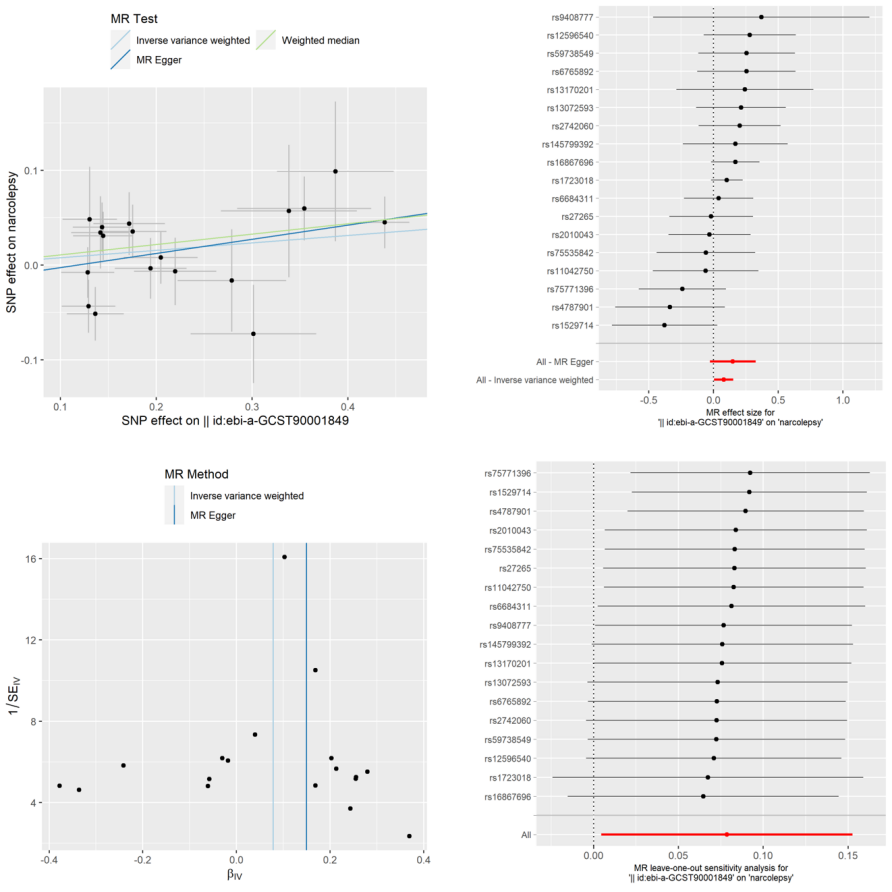


Figure S11. Scatter, Forest, Funnel, and Leave-one-out plots of genetically predicted CD3 on CD4+ T cell on effects NT1.


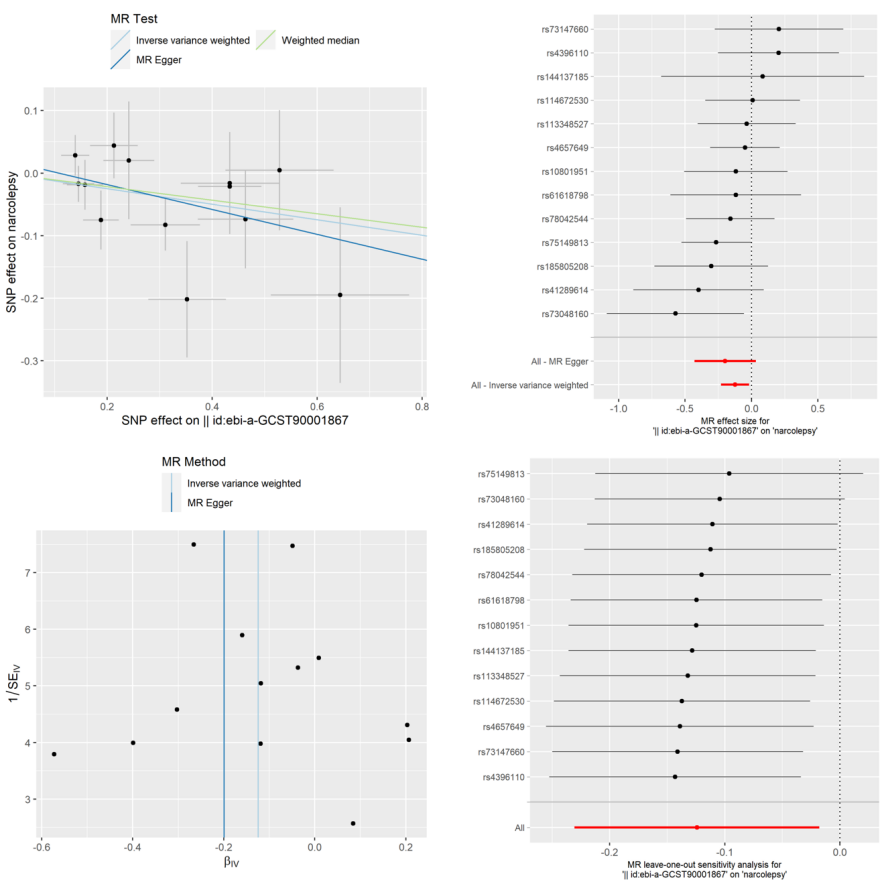


Figure S12. Scatter, Forest, Funnel, and Leave-one-out plots of genetically predicted HVEM on naive CD8+ T cell effects on NT1.


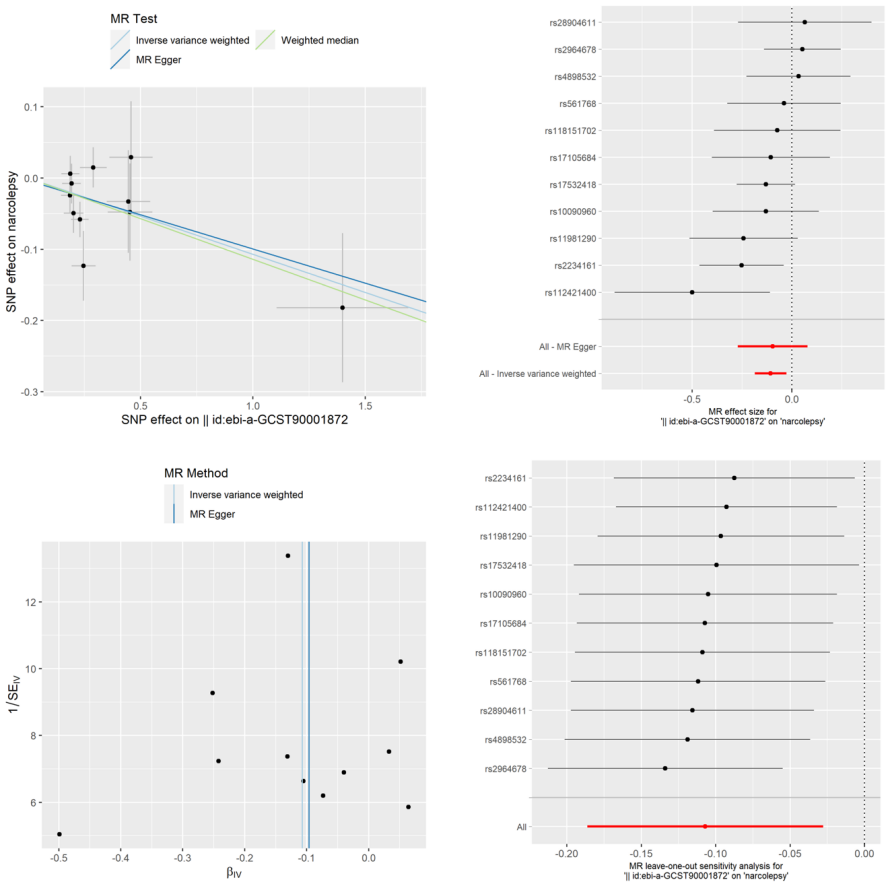


Figure S13. Scatter, Forest, Funnel, and Leave-one-out plots of genetically predicted CD28 on CD45RA+ CD4+ T cell effects on NT1.


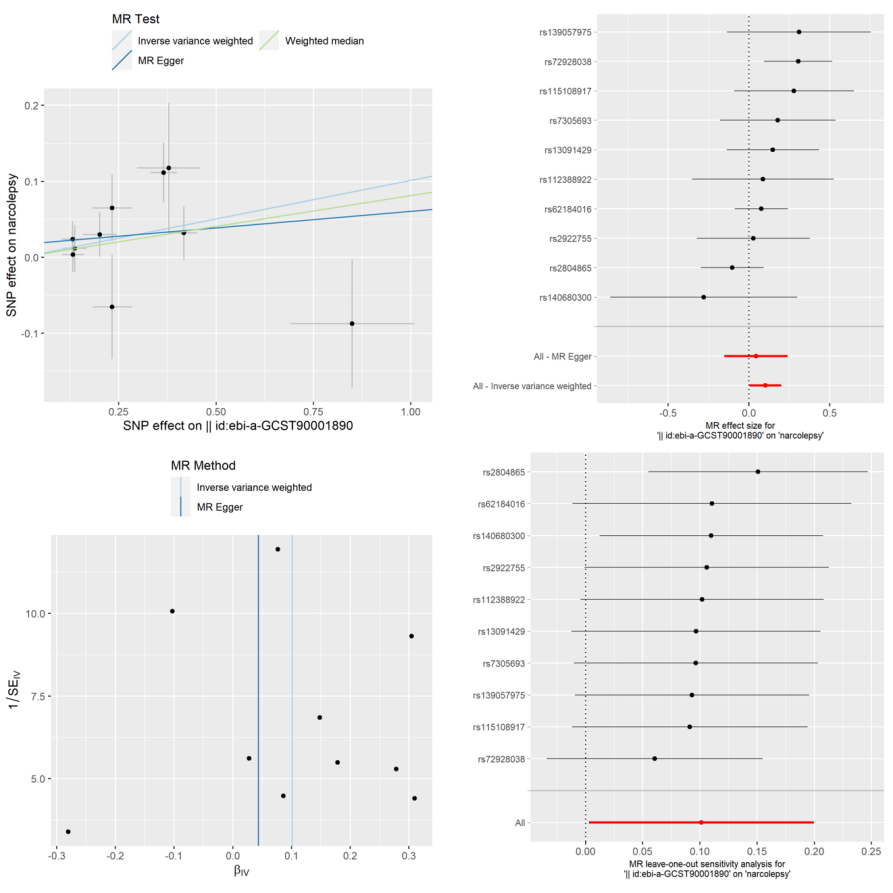


Figure S14. Scatter, Forest, Funnel, and Leave-one-out plots of genetically predicted CD28 on CD28+ CD45RA+ CD8+ T cell effects on NT1.


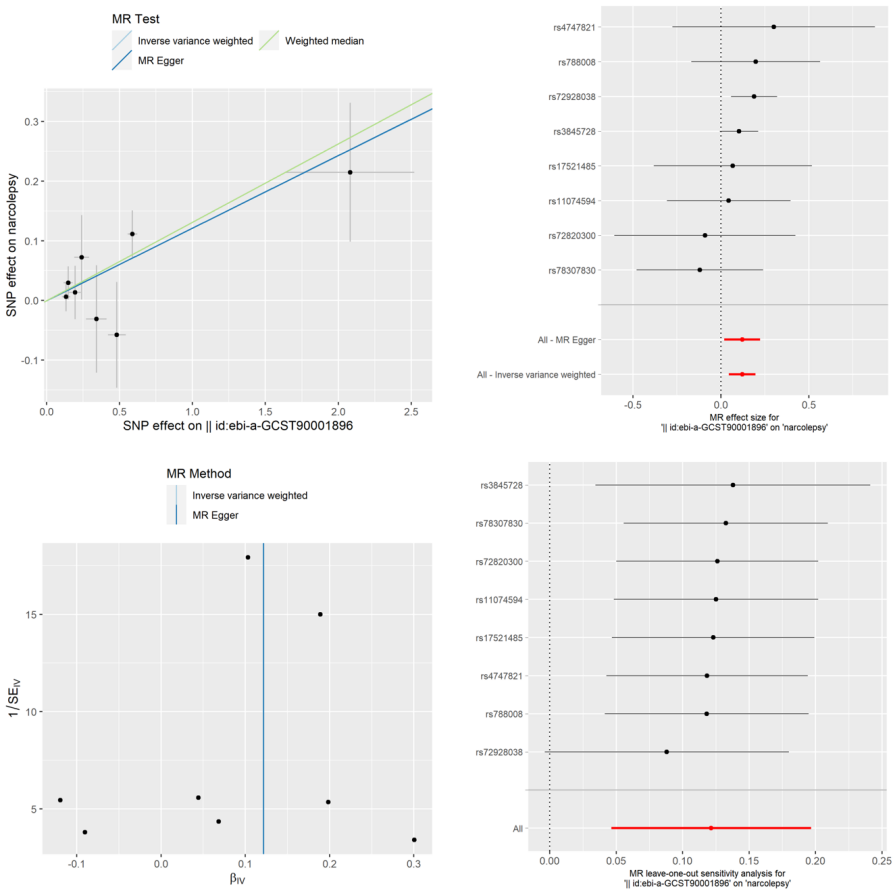


Figure S15. Scatter, Forest, Funnel, and Leave-one-out plots of genetically predicted CCR7 on naive CD8+ T cell effects on NT1.


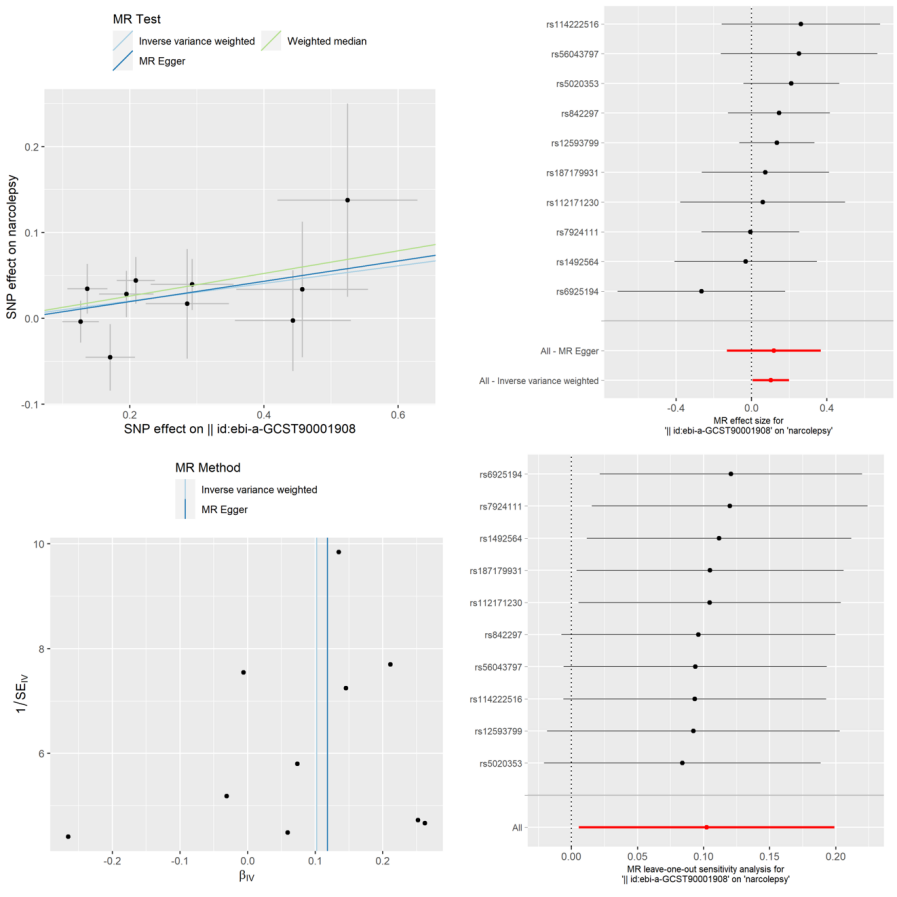


Figure S16. Scatter, Forest, Funnel, and Leave-one-out plots of genetically predicted CD45 on HLA DR+ CD4+ T cell effects on NT1.


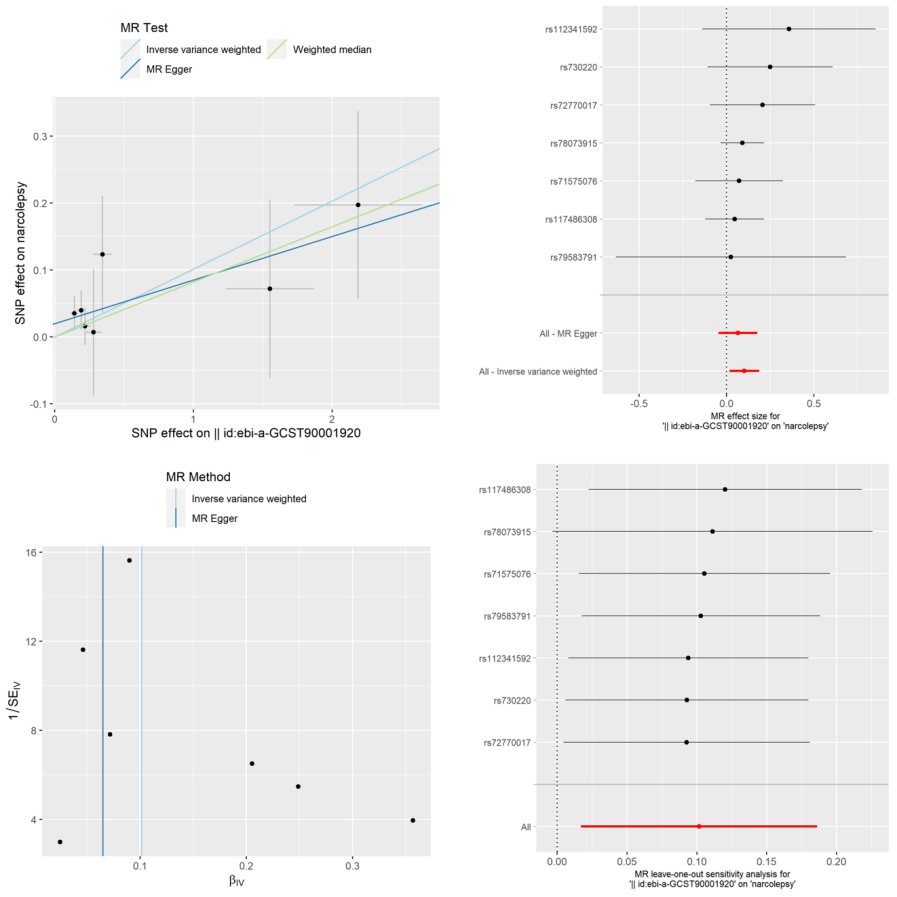


Figure S17. Scatter, Forest, Funnel, and Leave-one-out plots of genetically predicted CD127 on CD45RA- CD4 not regulatory T cell effects on NT1.


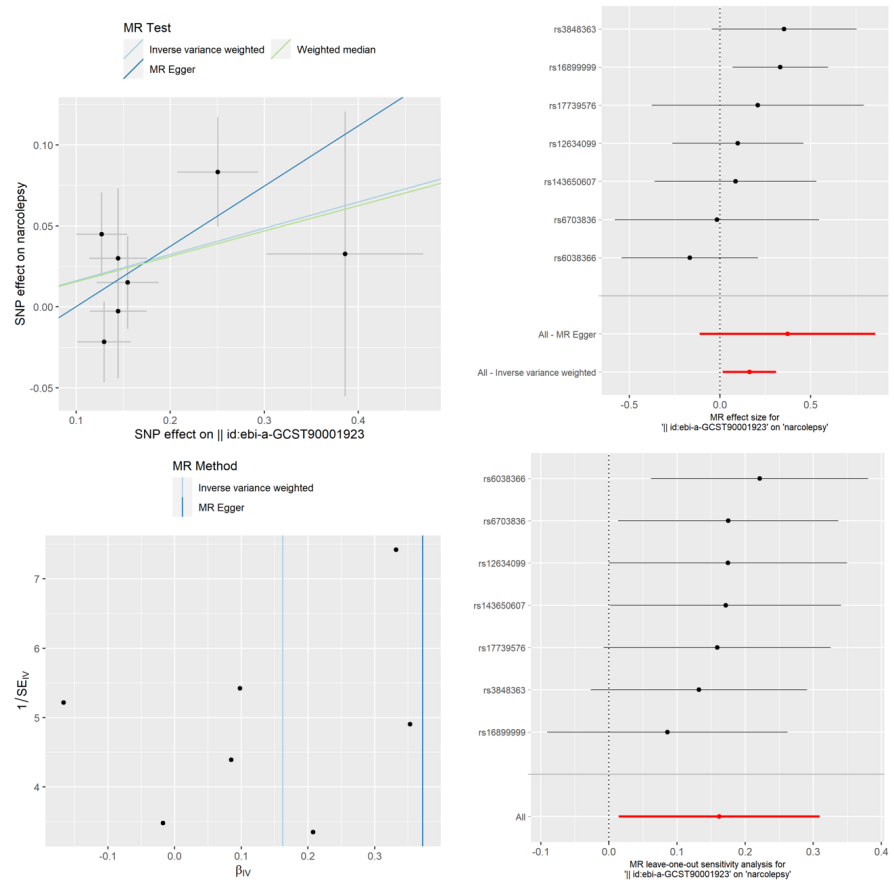


Figure S18. Scatter, Forest, Funnel, and Leave-one-out plots of genetically predicted CD127 on CD28+ CD4+ T cell effects on NT1.


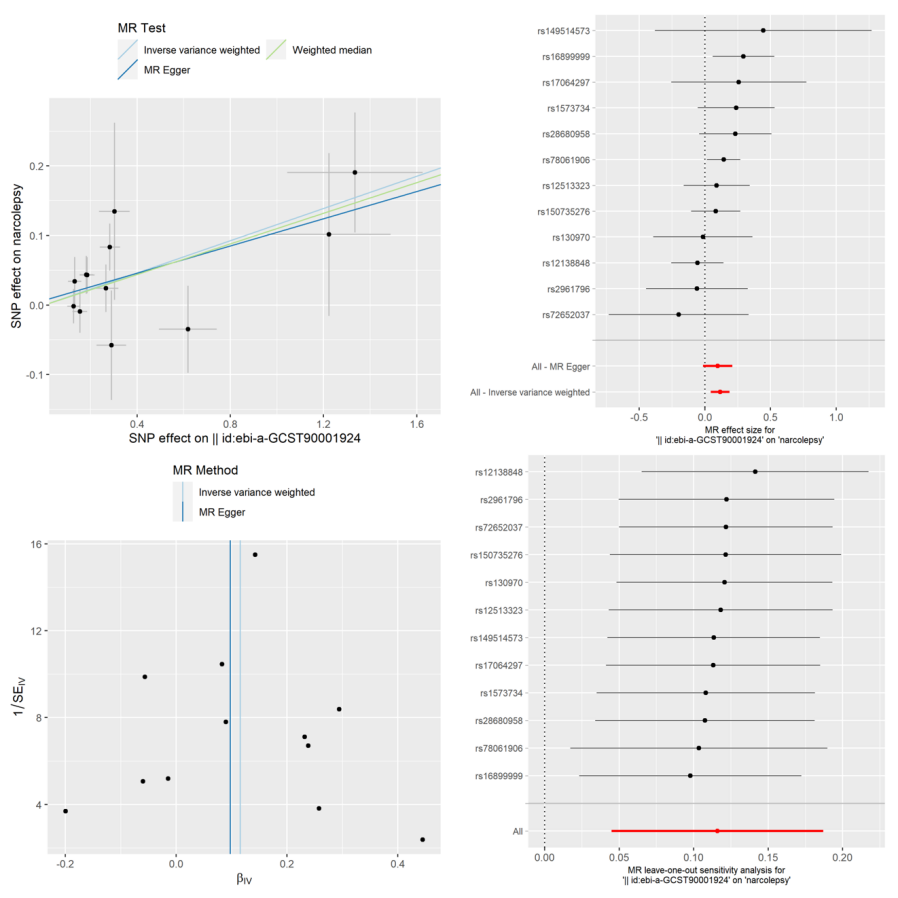


Figure S19. Scatter, Forest, Funnel, and Leave-one-out plots of genetically predicted CD127 on CD28- CD8+ T cell effects on NT1.


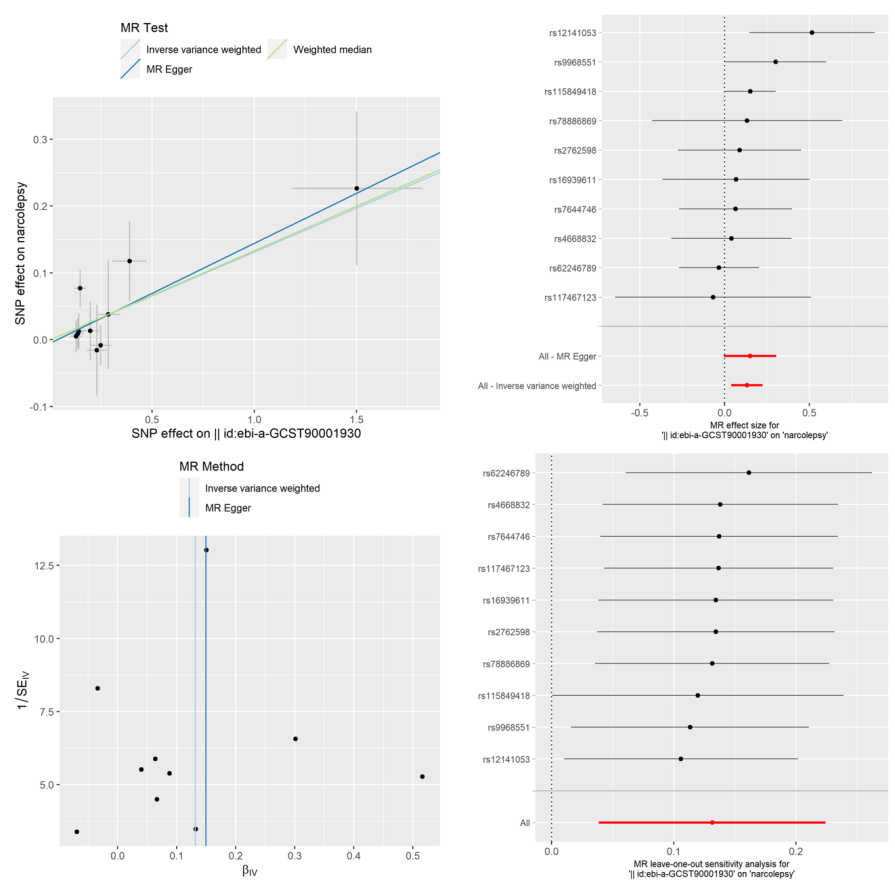


Figure S20. Scatter, Forest, Funnel, and Leave-one-out plots of genetically predicted CD25 on CD45RA- CD4 not regulatory T cell effects on NT1.


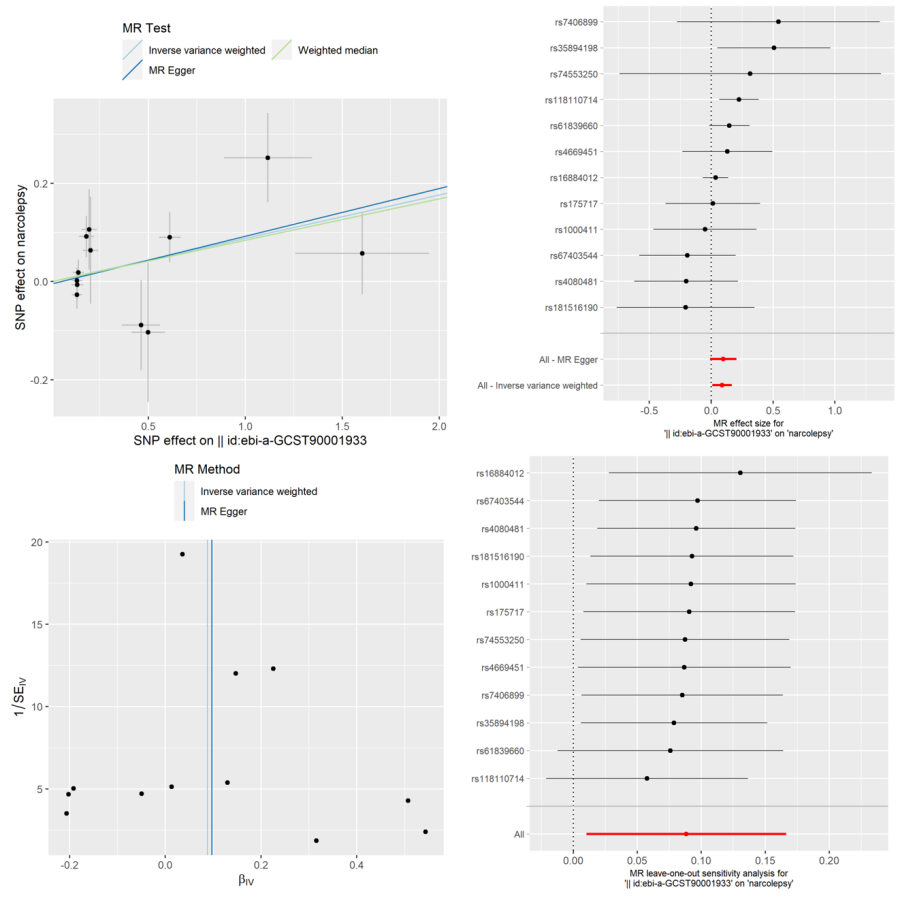


Figure S21. Scatter, Forest, Funnel, and Leave-one-out plots of genetically predicted CD8 on Effector Memory CD8+ T cell effects on NT1.

An outlier (rs17201560) was excluded


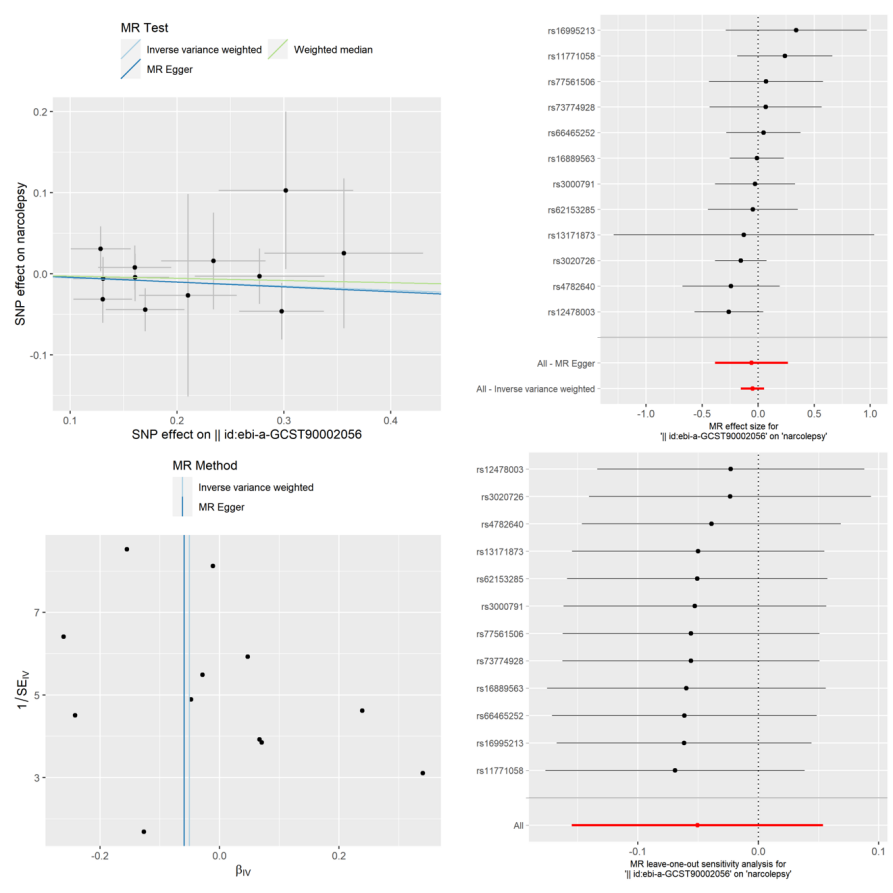


Figure S22. Scatter, Forest, Funnel, and Leave-one-out plots of genetically predicted CD8 on Terminally Differentiated CD8+ T cell effects on NT1.

Two outliers (rs114373132, rs960502) were excluded


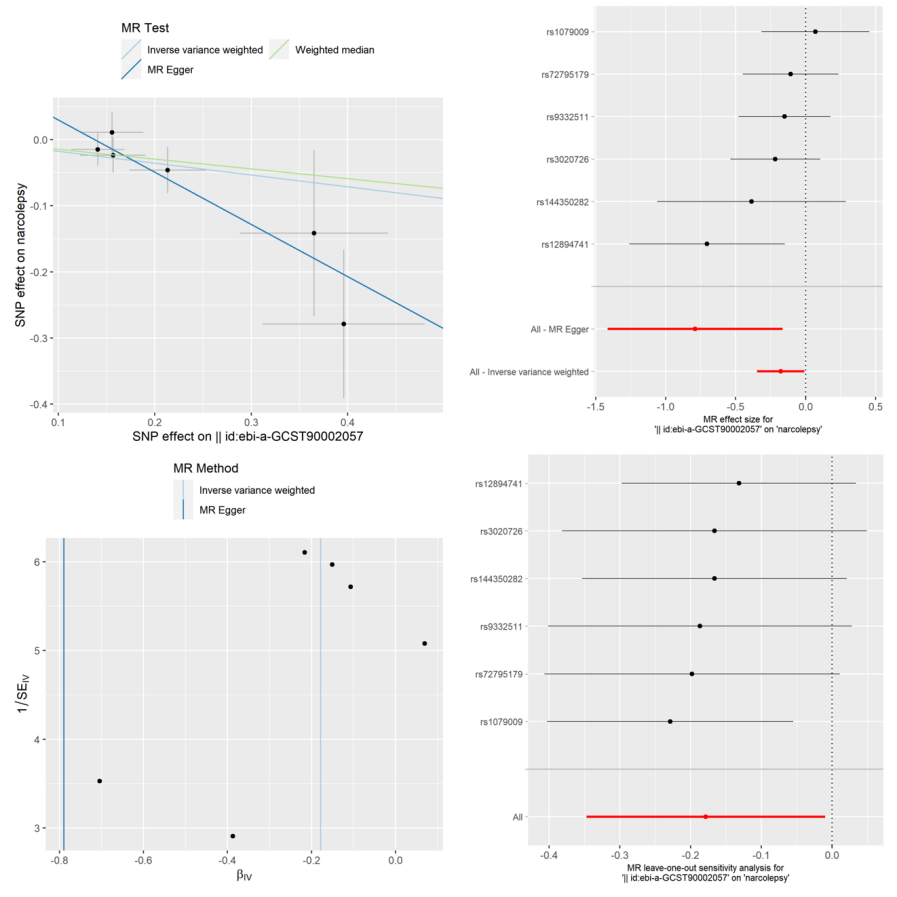


Figure S23. Scatter, Forest, Funnel, and Leave-one-out plots of genetically predicted CD8 on HLA DR+ CD8+ T cell effects on NT1.


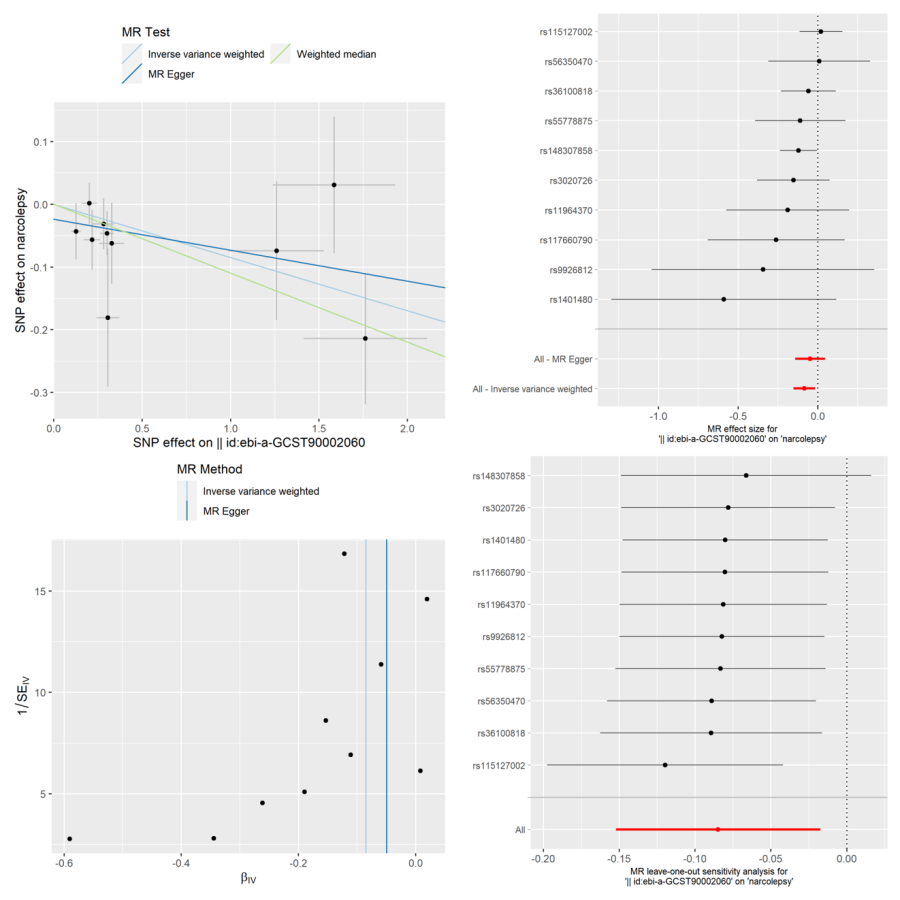

Supplement: Supplementary file 1 [file Data_Sheet_1.docx]
